# Predictive performance of four frailty screening tools in community-dwelling elderly

**DOI:** 10.1186/s12877-017-0633-y

**Published:** 2017-11-10

**Authors:** Bienvenu Bongue, Aurélie Buisson, Caroline Dupre, François Beland, Régis Gonthier, Émilie Crawford-Achour

**Affiliations:** 1 Département de Gérontologie Clinique, CHU de Saint Etienne, Hôpital de la Charité, 44 rue Pointe Cadet, 42000 Saint-Etienne, France; 2 Centre Technique d’Appui et de Formation des Centres d’Examens de Santé (CETAF), 67-69 Avenue de Rochetaillée, 42100 Saint-Etienne, France; 3 SOLIDAGE, McGill University - Université de Montréal Research Group on Frailty and Aging, Centre for Clinical Epidemiology, Jewish General Hospital, Montreal, Canada; 4 EA 4607, Laboratoire SNA-EPIS, Université Jean Monnet, Saint-Étienne, France

**Keywords:** Frailty, Elderly, Older persons, Older people, Screening tools, Screening instruments, Predictive performance

## Abstract

**Background:**

This study compares the performance of four frailty screening tools in predicting relevant adverse outcome (disability, institutionalization and mortality) in community-dwelling elderly.

**Methods:**

Our study involved a secondary analysis of data from the FréLE cohort study. We focused on the following four frailty screening tools: the abbreviated Comprehensive Geriatric Assessment (aCGA), the Groningen Frailty Indicator (GFI), the Vulnerable Elders Survey-13 (VES-13) and the Fried scale. We used the Barberger-Gateau scale to assess disability. For comparison, we determined the capacity of these tools to predict the occurrence of disability, institutionalization or death using the receiver operating characteristic (ROC) curve. We also determined the threshold at which an optimal balance between sensitivity and specificity was reached. Odds ratios (ORs) were calculated to compare the risk of adverse outcome in the frail versus non-frail groups.

**Results:**

In total, 1643 participants were included in the mortality analyses; 1224 participants were included in the analyses of the other outcomes (74.5% of the original sample). The mean age was 77.7 years, and 48.1% of the participants were women. The prevalence of frailty in this sample ranged from 15.0% (Fried) to 52.2% (VES-13). According to the Barberger-Gateau scale, 643 (52.5%) participants were fully independent; 392 (32.0%) were mildly disabled; 118 (9.6%) were moderately disabled; and 71 (5.8%) were severely disabled. The tool with the greatest sensitivity for predicting the occurrence of disability, mortality and institutionalization was VES-13, which showed sensitivities of 91.0%, 89.7% and 92.3%, respectively. The values for the area under the curve (AUC) of the four screening tools at the proposed cut-off points ranged from 0.63 to 0.75. The odds (univariate and multivariate analysis) of developing a disability were significantly greater among the elderly identified as being frail by all four tools.

**Conclusion:**

The multivariate analyses showed that the VES-13 may predict the occurrence of disability, mortality and institutionalization. However, the AUC analysis showed that even this tool did not have good discriminatory ability. These findings suggest that despite the high number of frailty screening tools described in the literature, there is still a need for a screening tool with high predictive performance.

**Electronic supplementary material:**

The online version of this article (10.1186/s12877-017-0633-y) contains supplementary material, which is available to authorized users.

## Background

Various definitions of frailty have been described in the literature. Clegg et al. [1] defined frailty as a state of vulnerability to poor resolution of homeostasis following a stressor and suggested that it was independently associated with important adverse outcomes. In an attempt to operationalize and standardize the definition of frailty, Fried et al. [2] used data from the Cardiovascular Health Study (CHS) and proposed a phenotype of frailty (CHS index) in which 3 or more of the following 5 components were present: unintentional weight loss, self-reported reduced energy level, reduced grip strength, slowed gait speed, and low level of physical activity. In contrast to the Fried et al. approach, Mitnitsky et al. [3] suggested an instrument based on an accumulation of deficits. Thus, two widely used frailty measures are the accumulation of deficits model, which uses the Frailty Index (FI) to characterize frailty as a state, and the Fried model, which describes frailty as a medical syndrome. Despite the substantial amount of work published in the past decade, a clear consensus of the definition of frailty has not emerged [4].

Although the conceptual definition of fragility remains to be established, current consensus suggests that frailty is potentially reversible [5]. Consequently, establishing a risk stratification system for frailty could be relevant in differentiating patients who would benefit from, would not benefit from, or would be harmed by an intervention. Clegg et al. [1] suggested that the most evidence-based process for detecting and grading the severity of frailty is the full Comprehensive Geriatric Assessment (CGA). The CGA is defined as a multidisciplinary diagnostic and intervention process that identifies the physical, cognitive, environmental, psychosocial and socioeconomic components that influence older adult health. However, application of this assessment is a resource-intensive process. An equally reliable but more efficient and responsive method for routine care is urgently needed. Given the increasing population of older adults, general practitioners (GPs) are in need of a frailty screening tool that is simple, easy to use, time-efficient and reliable. The results of this screening tool could determine the need for a full CGA, and inform the design of appropriate interventions [6].

Compared to non-frail elderly, the frail elderly have a higher risk of disability, falls, hospitalization, institutionalization, and death [7]. Several screening tools for frailty have been described in the literature [8, 9]; Buta et al. identified [10] 67 frailty instruments. However, in addition to the gap in the literature regarding the usefulness of many of these tools, their predictive performance remains unknown. Few studies have focused on the performance of these instruments [8, 11–13]. In a recent study, Daniels et al. [14] investigated the predictive validity of the Groningen Frailty Indicator (GFI), the Tilburg Frailty Indicator (TFI) and the Sherbrooke Postal Questionnaire (SPQ) in the development of disability. The study concluded that although all three of these instruments had the potential to identify older people at risk of frailty, the predictive performance was not sufficient.

This study compares the predictive performance of four frailty screening tools for relevant adverse outcome (development of disability, institutionalization and mortality) in community-dwelling elderly.

## Methods

### Design and study population

Data from the FRéLE study (Fragilité: une étude longitudinale de ses expressions) were used in this study. The aim of the FRéLE study was to identify the profiles and predictors of frailty among community-dwelling elderly, and the effect of these associated predictors on health and use of health and social services. This study has previously been described by Galand et al. [15]. The FRéLE study was a stratified multi-site observational longitudinal study with a two-year follow-up of community-dwelling elderly aged 65 years or older. The sample was stratified to ensure an equal number of people in crossed categories by age, sex and locale. At baseline, 1643 community-dwelling elderly living at home were included from the following three settings: metropolitan (CSSS Saint-Laurent-Bordeaux-Cartierville-Montréal), urban (CSSS Institut universitaire de gériatrie de Sherbrooke [CSSS-IUGS]) and semi-urban region (CSSS Des Érables). Five phases of data collection were utilized: three longer face-to-face interviews spaced at one year intervals, and two shorter telephone interviews to track and subjectively assess changes in health status.

The collected data included: socio-demographic variables (age, sex, marital status, social network, income, education, etc.); lifestyle (sleep patterns, alcohol and tobacco use); health status (frailty, comorbidities, cognitive status, depression, obesity, physiological impairments (sight, hearing, or lower-limb disability), and self-rated health); functional disabilities (Katz index (activities of daily living (ADL)), Lawton scale (instrumental ADL (IADL)), urinary and fecal incontinence); social network and support (help received, relationships with family and friends); and socio-psychosocial characteristics (locus of control, and satisfaction with life). For mortality, 1643 participants were included in the analyses. For institutionalization and disability, 1224 participants were included in the analyses.

### Materials

Our study involved a secondary analysis of data from the FréLE cohort. We focused on the following four frailty screening tools, which are commonly used in community-dwelling care: the abbreviated Comprehensive Geriatric Assessment (aCGA) [16]; the Vulnerable Elders Survey (VES)-13 [17]; the GFI [18]; and the Fried scale [2]. We chose these tools because of their usefulness and previous use, and because they were easily adapted using our database. Also, they are frequently cited in the literature. For example, Buta et al. [10] found that the VES-13 had been cited more than 200 times. Fried’s phenotype is a definition of frailty that has been used differently in previous epidemiological studies. For practical operationalization, we used proxy data to build our scales (see Additional file 1). This ensured that the data obtained from the FréLE study would allow us to construct our five tools (including the Barberger-Gateau scale for assessing disability).

### Frailty screening tools

- The aCGA consists of 15 questions covering three domains: functional status (seven questions on ADL and IADL); cognitive status (four questions from the Mini-Mental State Examination (MMSE)); and depression (four questions from the Geriatric Depression Scale (GDS)-15). A cut-off value for each domain was identified to indicate whether a more elaborate assessment was needed: namely, scores of > = 1 for ADL and IADL; ≤6 for the MMSE; and ≥2 for the GDS-4. Further assessment of frailty was needed if a positive score was identified in one of the aCGA domains [16]. In this study, the aCGA was only one of other measures used.
- The VES-13 was specifically developed to identify community-dwelling vulnerable elderly at risk for functional decline. This tool includes questions about age, self-rated health, physical fitness and the need for assistance with activities. It consists of 13 questions and has a maximum score of 10 points. We used the original cut-off value of ≥3 as an indication of frailty [17].
- The GFI is a screening tool used to determine the level of frailty. It consists of fifteen items and focuses on the loss of function and resources in four domains: physical (nine items); cognitive (one item); social (three items); and psychological (two items). Most items are answered with responses of ‘yes’ or ‘no’. The option ‘sometimes’ is added for cognitive and psychosocial items. Scores on the GFI range from zero to fifteen. The original cut-off value of ≥4 was used to indicate frailty [18].
- The Fried scale requires the measurement of only five variables, namely, weight loss, exhaustion, grip strength, gait speed and physical activity. The original cut-off value of ≥3 was used to indicate frailty, with scores of 1–2 indicating pre-frailty, and the absence of criteria indicating the absence of frailty [2]. A comparison between the FRéle-Fried scale and the original tool was previously presented by Galand et al. [15]

### Outcome measures

- Disability was defined according to the hierarchical disability scale proposed by Barberger-Gateau [19]. This tool includes 16 activities from three disability scales: five ADL items (bathing, dressing, going to the toilet, transferring and feeding); five IADL items (ability to use the telephone, shopping, mode of transportation, responsibility for own medication, ability to handle finances); three activities that were added to assess women (food preparation, housekeeping and laundry); and three items from the Rosow and Breslau functional health scale. The hierarchical disability scale proposed by Barberger-Gateau enables the identification of four levels of disability with increasing severity: full independence (absence of restriction in all three scales); mild disability (only mobility restriction); moderate disability (mobility and IADL restriction); and severe disability (mobility, IADL and ADL restriction) [19]. This hierarchical disability scale can be used to describe the progression of disability over time in elderly community dwellers. In this study, we defined disability as being moderately disabled or severely disabled. Thus, the event of interest was the occurrence of moderate disability (mobility and IADL restriction) or severe disability (mobility, IADL and ADL restriction) during the two-year follow-up period.
- Mortality data based on death registries were provided by the Institut de la statistique du Québec (ISQ – Quebec Institute of Statistics).
- Data on institutionalization were obtained from the Régie de l’assurance maladie du Québec (RAMQ – The organization responsible for public health insurance payment to providers in Québec). The RAMQ is the government health insurance board in the province of Quebec, Canada.

### Statistical analysis

Statistical analyses were conducted using three steps.

First, descriptive characteristics of the studied variables were generated using univariate analysis. For quantitative variables, the distribution of variables was characterized using the usual parameters (mean, median, mode, minimum, maximum, confidence interval around the mean, standard deviation). For qualitative variables, frequencies were calculated. Comparisons between the groups of subjects who were and were not included were performed using parametric and non-parametric tests according to variable distribution. Significance was indicated at the 5% level. The prevalence of frailty was calculated for each tool based on the cut-off provided by the relevant literature.

Second, the area under the receiver operating characteristic (ROC) curve was used to determine the capacity of each clinical test to predict mortality, institutionalization and disability, and to determine the threshold at which an optimal balance between sensitivity and specificity was obtained. The value of the area under the curve (AUC) can range from 0 to 1, with values over 0.8 indicating good predictive accuracy [20].

Third, odds ratios (ORs) were calculated to investigate the relationship between frailty, disability, institutionalization and mortality. ORs adjusted for age and sex were calculated using logistic regression models. For mortality analyses, we performed Cox regression model analyses. Statistical analyses were performed using SAS software (version 9.3; SAS Institute Inc., Cary, NC).

## Results

In total, 1643 participants were included in the mortality analyses; 1224 participants were included in the analyses of the other outcomes (74.5% of the original sample). Thus, after two years of follow-up, 419 subjects (146 deceased and 273 lost to follow-up) were excluded from analysis of disability and institutionalization. The mean age was 77.7 years. Those who died or had been lost to follow-up were mostly men (54.7%) and were older (81.7 years versus 77.7 years) and had lower annual incomes than those who remained in the analysis.

According to the Barberger-Gateau hierarchical scale, 643 (52.5%) participants were fully independent; 392 (32.0%) were mildly disabled; 118 (9.6%) were moderately disabled; and 71 (5.8%) were severely disabled. In total, after the two-year follow-up, 176 participants were newly disabled, 65 participants were moderately disabled, and 111 participants were severely disabled. Various transitional states were observed during the study period (see Additional file 2).

The prevalence of frailty in this sample ranged from 15.0% (Fried) to 52.2% (VES-13). Fifty-nine percent of the participants reported that they had been diagnosed with three or more chronic diseases. Table 1 summarizes the characteristics of the participants at T0 and T2 and of non-responders at T2, as well as the prevalence of frailty according to the various tools.

**Table 1:** Characteristics of participants at T0

	FréLE original sample (*n* = 1643)		Follow-up at T2 (1224)		Deceased (146) and lost to follow-up (273)		*p*
Sex							
Male	818	49.8%	589	48.1%	229	54.7%	0.021
Female	825	50.2%	635	51.9%	190	45.3%	
Mean age (SD)	78.7 (7.9)		77.7 (7.7)		81.7 (8.0)		0.000
Marital status							
With partner, married	895	54.5%	679	55.5%	216	51.6%	0.082
Widowed	460	28.0%	325	26.6%	135	32.2%	
Single	288	17.5%	220	18.0%	68	16,2%	
Institutionalization							
No	1580	96.2%	1186	96.9%	394	94.3%	0.015
Yes	62	3.8%	38	3.1%	24	5.7%	
Annual Income							
$0–20,000	724	44.9%	518	43.8%	196	48.3%	0.003
$20,001–40,000	570	35.4%	416	34.5%	154	37.9%	
$40,001 or more	317	19.7%	261	21.7%	56	13.8%	
Health status							
Number of chronic diseases							
0–2	674	41.0%	515	42.1%	159	37.9%	0.019
3–5	726	44.2%	523	42.8%	203	48.4%	
6–8	210	12.8%	155	12.7%	55	13.1%	
9 or more	32	1.9%	30	2.5%	2	0.5%	
^2^Neurological diseases							
Yes	31	1.9%	19	1.6%	12	2.9%	0.089
No	1611	98.1%	1204	98.4%	407	97.1%	
Cancer							
Yes	141	8.6%	97	7.9%	44	10.5%	0.105
No	1501	91.4%	1126	92.1%	375	89.5%	
Barberger-Gateau disability scale							
Full independence	788	48.0%	643	52.5%	145	34.6%	0.000
Mild disability	527	32.1%	392	32.0%	135	32.2%	
Moderate disability	209	12.7%	118	9.6%	91	21.7%	
Severe disability	119	7.2%	71	5.8%	48	11.5%	
Fried’s scale							
Normal	634	38.6%	530	43.3%	104	24.8%	0.000
Pre-frail	709	43.2%	511	41.7%	198	47.3%	
Frail	300	18.3%	183	15.0%	117	27.9%	
VES-13							
Normal	694	42.2%	585	47.8%	109	26.0%	0.000
Frail	949	57.8%	639	52.2%	310	74.0%	
GFI							
Normal	1086	66.1%	846	69.1%	240	57.3%	0.000
Frail	557	33.9%	378	30.9%	179	42.7%	
aCGA							
Normal	742	45.2%	604	49.3%	138	32.9%	0.000
Frail	901	54.8%	620	50.7%	281	67.1%	

*P* = difference between participants at T2 and non-responders at T2 (lost to follow-up or died)

*GFI* Groningen Frailty Indicator, *aCGA* abbreviated Comprehensive Geriatric Assessment, *VES-13* Vulnerable Elders Survey

Table 2 depicts the comparison of the diagnostic value of the four tools. The tool with the greatest sensitivity for predicting the occurrence of disability, mortality and institutionalization was VES-13, which showed sensitivities of 91.0%, 89.7% and 92.3%, respectively; however, the Fried scale had good specificities (Table 2). The values of the AUC for all four screening tools were between 0.63 and 0.75 (Table 2) at the cut-off points described in the literature.

**Table 2:** Diagnostic values of various screening tools for the development of disabilities, mortality and institutionalization

	Disability according to Barberger-Gateau (*n* = 1224)				
	Cut-off	Sensitivity (%)	Specificity (%)	AUC	
aCGA	≥1	87.5	60.2	0.74	
GFI	≥4	55.9	76.5	0.66	
Fried’s scale	≥3	43.7	93.5	0.68	
VES-13	≥3	91.0	59.3	0.75	
	Mortality (n = 1643)				
	Cut-off	Sensitivity (%)	Specificity (%)	AUC	
aCGA	≥1	74.5	48.1	0.61	
GFI	≥4	52.4	68.8	0.61	
Fried’s scale	≥3	41.5	85.2	0.69	
VES-13	≥3	87.7	46.7	0.67	
	Institutionalization (*n* = 1223)				
	Cut-off	Sensitivity	Specificity	AUC	
aCGA	≥1	84.6	51.3	0.68	
GFI	≥4	63.1	70.9	0.67	
Fried’s scale	≥3	40.0	86.4	0.63	
VES-13	≥3	92.3	50.1	0.71	

*AUC* Area under the curve, *GFI* Groningen Frailty Indicator, *aCGA* abbreviated Comprehensive Geriatric Assessment, *VES-13* Vulnerable Elders Survey

The odds (univariate and multivariate analysis) of developing a disability were significantly greater among the elderly identified as being frail by all four tools. The aCGA, was not associated with mortality (HR: 1.2 [0.8–1.7]) and institutionalization (OR: 2.1 [0.9–4.4]). Table 3 summarizes relationships between disability, mortality, institutionalization and the studied frailty screening tools.

**Table 3:** Odds ratios before and after adjustment for age and sex

		Disability according to Barberger-Gateau (*n* = 1224)		^a^Mortality (*n* = 1643)		Institutionalization (*n* = 1223)	
		*p*	OR (95% CI)	*p*	^b^HR (95% CI)	*p*	OR (95% CI)
Univariate	aCGA	0.000	6.5 (4.4–9.6)	0.000	1.8 (1.3–2.5)	0.000	3.8 (1.8–7.7)
	GFI	0.000	3.8 (2.7–5.2)	0.000	1.9 (1.4–2.4)	0.000	3.4 (2.0–5.8)
	Fried’s scale	0.000	7.5 (5.1–10.9)	0.000	2.3 (1.7–3.1)	0.001	2.6 (1.5–4.6)
	VES-13	0,000	6.7 (4.1–10.9)	0.000	3.5 (2.1–5.6)	0.000	8.1 (2.9–22.4)
Multivariate	aCGA	0.000	3.6 (2.4–5.5)	0.356	1.2 (0.8–1.7)	0.055	2.1 (0.9–4.4)
	GFI	0.001	1.8 (1.3–2.6)	0.069	1.3 (0.9–1.8)	0.009	2.1 (1.2–3.8)
	Fried’s scale	0.000	3.5 (2.3–5.2)	0.001	1.7 (1.3–2.4)	0.243	1.4 (0.8–2.5)
	VES-13	0.000	3.2 (1.9–5.4)	0.000	2.7 (1.6–4.5)	0.004	4.6 (1.6–13.2)

^a^COX regression (adjusted on age and sex); ^b^ *HR* Hazard Ratio

## Discussion

The aim of this study was to investigate the predictive performances of four frailty screening tools: the Fried scale, the Groningen Frailty Indicator (GFI), the Vulnerable Elders Survey (VES-13) and the abbreviated CGA (aCGA). Based on the AUCs and the multivariate analyses, the VES-13 appeared to be the most suitable tool to predict the occurrence of disability, death and institutionalization. This screening tool showed a high sensitivity for disability (91.0%), mortality (89.7%) and institutionalization (92.3%), but the specificities were relatively low (59.3%, 45.4% and 50.1% for disability, mortality and institutionalization, respectively). Our results were in accordance with those of previous studies. Daniels et al. [14] assessed three frailty screening tools and found that they performed poorly. In a recent review, Dent et al. [21] concluded that there is currently no single perfect frailty measurement. Some tools are better for population-level frailty screening, whereas others are best suited for clinical screening or assessment.

Many studies have examined the risk factors for institutionalization. Aguero-Torres et al. [22] found that regarding age-related conditions, dementia and cognitive impairment were the main contributors to institutionalization in the elderly, independent of individual functional status and social network. Our findings showed that the Fried scale was the most specific (86.4%) in terms of predicting institutionalization, but the sensitivity was low (40.0%) and in addition, the association was not significant in the regression analysis. This result suggests that the Fried scale is not a suitable screening tool in identifying older persons for institutionalization. This result could be explained by the fact that some domains such as cognition and isolation, which are important risk factors for institutionalization, are not included in the Fried tool. To our knowledge, our study is one of the first to show this finding.

Regarding the AUC values, of these four tools, none had good predictive capacity (AUC between 0.63 and 0.75). Long et al. suggest that values over 0.8 indicate good predictive accuracy [20]. The VES-13 showed acceptable discriminating ability for institutionalization (AUC = 0.71) and disability (AUC = 0.75) but the aCGA had acceptable performance for disability only (AUC = 0.74). Our results are in accordance with those of other researchers. In a review of the predictive accuracy of major frailty scores, Pijpers et al. [23] concluded that these tests are not sensitive enough for screening and diagnostic purposes. This finding was disappointing because there is a need for an easy, feasible, less time-consuming screening tool with good predictive accuracy in clinical practice.

The prevalence of frailty in this sample ranged from 15.0% (Fried) to 52.2% (VES-13). Our results are in accordance with those of previous studies [24–28]. In a recent study, Sutorius et al. [12] found that the prevalence rates of frailty ranged from 14.8% (Frailty Index) to 52.9% (Identification of Seniors at Risk-Primary Care (ISAR-PC)). For the Fried tool and the GFI, Theou et al. found similar results to our study [11]. In this study, we found a prevalence of 15.0% using the Fried scale. This result was similar to that found in the SHARE-FI75+ study (15.7%), which used the same tool [26]. In our study, the prevalence of frailty using the GFI was 33.9%, which was comparable to the rate found in a study conducted by Hoogendijk et al. [13] Regarding the Barberger-Gateau hierarchical scale of disability, Preres K and colleagues in the Paquid cohort (Personnes agées Quid, i.e., “what about older adults?”) found that the prevalence of full independence was 20.7%, the prevalence of mild disability was 42.9%, the prevalence of moderate disability was 29.5% and the prevalence of severe disability was 6.9% [29]. The difference between our study and that of Perez et al. may be explained by age; specifically, Perez et al. included older participants (mean age 80 years vs. 77.7 years in our study). The INSERM report [30] found that the prevalence of full independence was 43.1%, the prevalence of mild disability was 40.4%, the prevalence of moderate disability was 14.0% and the prevalence of severe disability was 2.5%. Our results were comparable. We found that 12.7% of participants were moderately disabled and that 7.3% of participants were severely disabled. The variations in prevalence that were found in this study based on the tool used suggest that tools measure different dimensions of frailty. No consensus exists about which domains should be included in the definition and operationalization of frailty, nor what the best measurement of frailty is [4]. However, many researchers believe that the definition of frailty should include domains such as cognition, mood, and other aspects of mental health in addition to physical functioning [7, 31]. The differences in prevalence found in this study could be explained by the lack of consensus on which domains should be included in the operational definition of frailty.

In the past decade, comparisons of the accuracy of frailty screening tools have been described in the literature [11, 12, 32–36]; however, the results have been conflicting. The findings of studies including older outpatients differ depending on the characteristics of the study population and the screening tools used. In their analysis of 102 individuals living at home in the Netherlands, Hoogendijk et al. [13] evaluated the following five screening tools for frailty: the GFI, PRISMA-7, the Fried scale, clinical judgment, and general practitioner (GP) assessment of health. The authors suggested that the PRISMA-7 was the most appropriate tool. However, this single-center study included a small number of participants who were recruited using a postal questionnaire. Daniels et al. [14] evaluated the following three tools: the GFI, TFI and SPQ. They did not find any one tool superior to another (AUC values between 0.54 and 0.67). The study included 430 people in the Netherlands from four GP practices who were recruited by a postal questionnaire and followed for one year. Changes in disability status were not considered in the analysis, and many participants were lost to follow-up. When comparing the accuracy of five simple tools used to identify frail older adults in primary care, Hoogendijk et al. reported that the GFI had the lowest AUC (0.64) [13]. Deckx et al. found comparable results [34]. These results were in accordance with our findings.

The relationship between frailty, disability, and mortality is well known. Fried et al. found that although individuals who are classified as frail can certainly have disabilities, multiple comorbid illnesses, and advanced age, frailty can occur even when these conditions are absent [37]. These authors also associated frailty with dependence, institutionalization and mortality [2, 37].

Our results show that the Fried tool was the most specific in terms of predicting institutionalization, but this finding was not significant in the regression analysis. This suggests that the Fried tool could be more suitable for assessment of institutionalized older persons than for screening of this population.

Although our results mostly reflected the same trends, our study found that the aCGA was not related to mortality or institutionalization and that the Fried tool was not associated with institutionalization. This result is very interesting because it shows that even when there is a link between frailty, disability and institutionalization, some frailty screening tools may not be appropriate for assessing mortality and institutionalization in community-dwelling older adults.

Our study had some limitations, and the results should thus be interpreted with caution. Because this study compared four frailty scales using data obtained from the same questionnaire, some criteria had to be modified prior to application. After the required adaptation, some tools may have retained more similarity to the original than others. Future studies should compare tools in their original form to determine whether findings are similar regarding their predictive performances. However, modifying the criteria for frailty is common, especially regarding the widely applied Fried scale [11, 12, 38, 39]. Direct comparisons between tools should also be made with caution, especially between those designed to be screening tools (e.g., the FRAIL scale) and those designed to be assessment tools (e.g., the FI) [40].

The VES-13 has been especially developed to identify older persons at risk of death or functional decline [17, 41]. This screening tool included questions about the ability to perform six physical and five functional activities, self-rated health, and age. Although the VES-13 is a widely used tool for measuring frailty, this screening tool has a major defect, in that it includes age. Disability and mortality are almost linearly correlated with age at old age. Perhaps this index would not be informative if age was not included.

Another limitation was the study population. The FréLE sample was stratified, with an equal number of people in crossed age, sex and city categories. This strategy helps in including hard to recruit very old persons in the sample, particularly elderly men, but requires weighting cases to enhance the external validity of the findings. Furthermore, the lack of continuous observations of disability during the two-year period was another limitation. Functional status was assessed at only two discrete times. Thus, episodes of increasing disability following recovery could have been missed. Similarly, disability status could not be assessed immediately before death in deceased subjects. In this study, we also assumed that a transition between two non-consecutive disability states involved an intermediate state, even if this pattern was not observed due to the discrete observation times. We concentrated on the occurrence of moderate and severe dependence, as these indicators have strong financial and social consequences and are considered to be reliably reversible. We then discounted this reversibility, as estimations of the probability of transition become very complicated in models that include reversibility between several states.

## Conclusion

The multivariate analyses showed that the VES-13 may predict the occurrence of death, disability and institutionalization. However, the AUC analysis showed that even this tool did not have good discriminatory ability. These findings suggest that despite the high number of frailty screening tools described in the literature, there is still a need for a screening tool with high predictive performance.

## Additional file

Additional file 1: Operationalization of FréLe data to build our scales. (DOCX 27 kb)
Additional file 2: Supplementary data. (DOCX 25 kb)

## Abbreviations

aCGA: Abbreviated comprehensive geriatric assessment
AUC: Area under the curve
GFI: Groningen Frailty Indicator
VES-13: Vulnerable Elders Survey
