# Supplementary material for: Predictive performance of four frailty screening tools in community-dwelling elderly
Source: BMC Geriatr. 2017 Nov 10;17:262. doi: 10.1186/s12877-017-0633-y (PMC5681791; doi:10.1186/s12877-017-0633-y)
Supplement: Additional file 1: — Operationalization of FréLe data to build our scales. (DOCX 27 kb) [file 12877_2017_633_MOESM1_ESM.docx]

Additional file

**Additional file 1: Operationalization of FréLe data to build our scales**

**Frieds’scale**

| Original Scale | Frêle Scale |
| --- | --- |
| In the last year, you lost more than10 pouns unintentionally (i.e., not due to dieting or exercise) ?  **No=0 ; Yes=1** | Loss ≥ 10% of weight at age 60  or  Loss ≥ 4 Kg during the last year  **No=0 ; Yes=1** |
| How often in the last week did you feel this way ? (a) I felt that everything i did was an effort ; (b) I could not going. Rarely or none of the time (<1 day)=0 some or little of the time (1-2 days=0 ; a moderate amount of the time (3-4 days)=1 ; most of the time=1 | First quintile on the "Vitalité" scale of SF36:  Vitality ≤ 46.9 |
| Minnesota Leisure Time Activity questionnaire : Walking, chores (modarately strenuous), mowing the lawn, raking, gardening, hiking, jogging, biking, exercise cycling, dancing, aerobics, bowling, golf, singles tennis, doubles tennis, racquetball, calisthenics, swimming.  **Men : Kcals/ week <383=1**  **Women : Kcals/ week <270=1** | First quintile on the *Physical Activity Scale for Elderly* (PASE)  *Women* : ≤ 28.2  *Men* : ≤ 33.5 |
| Walking Time/15 feet measurement  **Men :**  **Height ≤173 cm and time≥7 seconds=1**  **Height >173 cm and time≥6 seconds=1**  **Women :**  **Height ≤ 159 cm and time≥7 seconds=1**  **Height >159 cm and time≥6 seconds=1** | First quintile of walking speed over a distance of 4 meters  Women:  Speed ​​≤ 55 cm / s (size ≤ 156 cm)= 1  Speed ​​≤ 65 cm / s (size> 156 cm)=1  Men :  Speed ​​≤ 63 cm / s (size ≤170 cm)=1  Speed ​​≤ 69 cm / s (size> 170 cm)=1 |
| Handgrip strenght measure by dynamometer  **Men**  **BMI≤24 and strenght ≤29=1**  **BMI 24.1-26 and strenght ≤30=1**  **BMI 26.1-28 and strenght ≤30=1**  **BMI >28 and strenght ≤32=1**  **Women**  **BMI≤23 and strenght ≤17=1**  **BMI 23.1-26 and strenght ≤17=1**  **BMI 26.1-28 and strenght ≤18=1**  **BMI >29 and strenght ≤21=1** | Handgrip strenght measure by dynamometer (Kp)  *Women:*  *-≤34kP with BMI≤24.1=1*  *-≤35kP with BMI≤24,1≤BMI≤27,4=1*  *-≤35kP with BMI≤27.4≤BMI≤30.9=1*  *Men:*  *-≤47kP with BMI≤24.7=1*  *-≤53kP with BMI≤24,7≤BMI≤27.3=1*  *-≤53kP with BMI≤27,3≤BMI≤30,5=1*  *-≤54kP with BMI> 30.5=1* |

**The Groningen Frailty Indicator (GFI) scale**

| Original Scale | Frêle Scale |
| --- | --- |
| Is the patient able to carry out these tasks single handed without any help ? (The use of help resources such as walking stick, walking frame, Wheelchair , is considered independent) ; (1) Shopping ; (2) walking around outside (around the house or to the neighbors) ; (3) Dessing and undressing ; (4) Going to the toilet  **For each item, independent = 0 ; Dependent = 1** | Same as original |
| Does the patient experience problems in daily life due to poor vision ?  **No=0 ; Yes=1** | Can you read the newspapers well enough with your glasses or contact lenses  **No=0 ; Yes=1** |
| Does the patient experience problems in daily life due to being hard of hearing  **No=0 ; Yes=1** | Usually, can you hear what is said in a group conversation (with at least three other people) with the help of a hearing aid?  **No=0 ; Yes=1** |
| During the last 6 months has the patient lost a lot of weight unwillingly ? (3kg in 1 month or 6 kg in 2 months)  **No=0 ; Yes=1** | Loss ≥ 10% of weight at age 60  or  Loss ≥ 4 Kg during the last year  **No=0 ; Yes=1** |
| Does the patient take four or more different types of medecine ?  **No=0 ; Yes=1** | Same as original  **No=0 ; Yes=1** |
| Does the patient have any complaints about his/her memory or is the patient know to have a dementia syndrome  **No=0 ; Yes=1** | Do you feel you have more memory problems than the majority of people?  **No=0 ; Yes=1** |
| Does the patient sometimes experience an emptiness around him/her ? (2) Does the patient sometimes miss people around him/her (3) Does the patient sometimes feel abandoned ? (4) Has the patient recently felt downhearted or sad (5) Has the patient recently felt nervous or anxious  **For each item, No=0 sometimes and yes=1 (five possible points**) | Same as original |
| What mark does the patient give himself/herself for physical fitness (scale 0 to 10)  **7-10=0 ; 0-6=1** | In general, would you say your health is: Excellent = 0, Very good = 0, Good = 0, Fair =1 ; Poor = 0 |

**VES-13**

| **Original Scale** | **Frêle VES-13** |
| --- | --- |
| Q1 age  Score : 1 point for age 75-84  3 points for age ≥ 85 | Same as original |
| **Q2 :** In general, compared to other people your age, would you say that your health is :  Poor = 1, Fair = 1, good = 0 | Same as original |
| **Q3 :** How much difficulty, on average, do you have with the following physical activities   1. Stooping, crouching or kneeling ? 2. Lifting, or carrying objects as heavy as 10 pounds ? 3. Reaching or extending arms above shoulder level 4. Writing, or handling and grasping small objects 5. Walking a quarter of a mile ? 6. Heavy housework such as scrubbing floors or washing windows ?   ***Score : 1 point for each response in Q3a through f. Maximum of 2 points*** | Does your current state of health limit you in one of the following activities:  Same as original  In activities requiring significant physical effort, such as running, lifting heavy objects or practicing violent sports ?  Same as original  Can you read the newspapers well enough with your glasses or contact lenses  No=0 ; Yes=1  Walking more than a kilometer? a lot =0, a little = 0, not at all = 1  Same as original |
| **Q4 :** Because of your health or physical condition, do you have any difficulty :   1. Shopping for personal items (like toilet or medecines) 2. Managing money 3. Walking across the room ? 4. Doing light housework ? 5. Bathing or showering ?   ***Score : 4 points for one or more responses in Q3a through Q4e*** | Same as original |

***For the aCGA Scale the FréLe scale was the same as original**

|  | |  | Appendix 1 : Study population and transitional states (n=1643) | | | | | | | | | |  | |  |  |  |  |  |  |  |
| --- | --- | --- | --- | --- | --- | --- | --- | --- | --- | --- | --- | --- | --- | --- | --- | --- | --- | --- | --- | --- | --- |
|  | |  |  |  |  |  |  |  | |  | |  |  | |  |  |  |  |  |  |  |
|  | |  |  |  |  |  |  |  | |  | |  |  | |  |  |  |  |  |  |  |
| T0 | |  | 1315 (Fully independent or mildly disabled) | |  |  | **328 (Moderately disabled or severely disabled)** |  | |  |  | |  | |  |  |  |  |  |  |  |
|  | |  |  |  |  |  |  |  | |  |  | |  | |  |  |  |  |  |  |  |
|  | |  |  |  |  |  |  |  | |  |  | |  | |  |  |  |  |  |  |  |
|  | |  | 1077 (Fully independent or mildly disabled) | |  |  | **309 (Moderately disabled or severely disabled)** | | |  |  | | **191 lost to follow-up** | | |  |  | **66 deceased** | |  |  |
| T1 | | status at T0 | 1038 | **39** |  |  | 103 | **206** | |  |  | | 127 | | **64** |  |  | 47 | **19** |  |  |
|  | |  |  |  |  |  |  |  | |  |  | |  | |  |  |  |  |  |  |  |
|  | |  |  |  |  |  |  |  | |  |  | |  | |  |  |  |  |  |  |  |
|  | |  | 945 (Fully independent or mildly disabled) | | |  | **279 (Moderately disabled or severely disabled)** | | | |  | | **273 lost to follow-up** | | | |  | **146 deceased** | | | |
| T2 | | Status T1 | 892 | **39** | **14** |  | 79 | **193** | | **7** |  | | 75 | | **41** | **157** |  | 31 | **36** | **13** | **66** |
|  | |  |  |  |  |  |  |  | |  | |  |  | |  |  |  |  |  |  |  |
|  | |  |  |  |  |  |  |  | |  | |  | |  |  |  |  |  |  |  |  |
|  | |  |  |  |  |  |  |  | |  | |  | |  |  |  |  |  |  |  |  |
|  | |  |  |  |  |  |  |  | |  | |  | |  |  |  |  |  |  |  |  |
|  | For example:  Of the 945 participants fully independent or mildly disabled at T2,   - 892 were fully independent or mildly disabled at T1, - 39 were moderately disabled or severely disabled at T1, - 14 could not be reached at T1 but were seen at T2 | | | | | | | |  |  | |  | |  |  |  |  |  |  |  |  |
